# Marked Response in Microbial Community and Metabolism in the Ileum and Cecum of Suckling Piglets After Early Antibiotics Exposure

**DOI:** 10.3389/fmicb.2018.01166

**Published:** 2018-05-30

**Authors:** Miao Yu, Chunlong Mu, Chuanjian Zhang, Yuxiang Yang, Yong Su, Weiyun Zhu

**Affiliations:** ^1^Jiangsu Key Laboratory of Gastrointestinal Nutrition and Animal Health, Laboratory of Gastrointestinal Microbiology, College of Animal Science and Technology, Nanjing Agricultural University, Nanjing, China; ^2^National Center for International Research on Animal Gut Nutrition, Nanjing Agricultural University, Nanjing, China; ^3^Guangdong Key Laboratory of Animal Breeding and Nutrition, State Key Laboratory of Livestock and Poultry Breeding, Institute of Animal Science, Guangdong Academy of Agricultural Sciences, Guangzhou, China

**Keywords:** ileum, cecum, early antibiotics exposure, microbial community, microbial metabolites, suckling piglets

## Abstract

In modern swine husbandry systems, antibiotics have been used as growth promoters for piglets during suckling or weaning period. However, while early colonization of intestinal microbiota has been regarded crucial for the host’s later life performance and well-being, little is known about the impact of antibiotics on intestinal microbiota in suckling piglets. The present study aimed to investigate the effects of early antibiotics exposure on gut microbiota and microbial metabolism of suckling piglets. Sixteen litters of suckling piglets were fed a creep feed diet with (Antibiotic) or without (Control) antibiotics from postnatal days 7–23 (*n* = 8). The ileal and cecal digesta were obtained for microbial composition and microbial metabolites analysis. The results showed that the antibiotics significantly altered the bacterial community composition by decreasing (*P* < 0.05) the diversity and richness in the ileum. The antibiotics significantly reduced the abundance of *Lactobacillus* in both the ileum and cecum, increased the abundance of *Streptococcus*, unclassified Enterococcaceae, unclassified Fusobacteriales, and *Corynebacterium* in the ileum, and the abundance of unclassified Ruminococcaceae and unclassified Erysipelotrichaceae in the cecum. The antibiotics decreased (*P* < 0.05) ileal lactate concentration and cecal concentration of total short-chain fatty acids (SCFAs). But the antibiotics enhanced protein fermentation (*P* < 0.05) in the ileum and cecum, as ileal concentrations of putrescine and cadaverine, and cecal concentrations of isobutyrate, isovalerate, putrescine, cadaverine, spermine, and spermidine were significantly increased (*P* < 0.05). These results indicated that early antibiotics exposure significantly altered the microbial composition of suckling piglets toward a vulnerable and unhealthy gut environment. The findings provide a new insight on the antibiotics impact on neonates and may provide new framework for designing alternatives to the antibiotics toward a healthy practice for suckling piglets.

## Introduction

The gut microbes of mammals are integral to the prevention of infectious diseases, maintenance of intestinal morphology, nutrient digestion and metabolism, immune modulation of the host (Nicholson et al., 2005; Romick-Rosendale et al., 2009; Hooper et al., 2012). Immediately after birth, the piglet gut is rapidly colonized by a complex and diverse microbiota derived from the sow and environment (Konstantinov et al., 2006). As the pig grows, the gut microbiota becomes mature as reflected by highly diverse, stable and established microbial community. Our previous study demonstrated that the overall gut microbial community of the pig showed an age-dependent maturation, with weaning as an important event for the gut microbe succession (Bian et al., 2016). During the early life stages, the composition and diversity of the gut microbiota is dynamic and can be easily influenced by environmental conditions, such as the use of creep feed and medicines (antibiotics and vaccines), and by the exposure to pathogenic microorganisms (Cho et al., 2012; Schokker et al., 2014). Antibiotics have been used in feed as beneficial practice in suckling or weaning piglets to improve feed efficiency for many years in China and many other countries. Previous studies have shown that antibiotics in feed altered the gut microbial communities with detected shifts in bacterial functions and membership in weaning piglet feces (Allen et al., 2011; Looft et al., 2012) or growing pig intestine (Looft et al., 2014), suggesting an evident impact of antibiotics on the gut microbiota. However, while it is regarded that the early colonization of gut microbes is important for the host’s later life performance and well-being (Conroy et al., 2009; Saavedra and Dattilo, 2012; Hansen et al., 2013), little information is available about the impact of early life antibiotics exposure on gut microbiota of suckling piglets.

Antibiotics also influence microbial fermentation in the intestine. Gut microbes ferment carbohydrates to produce short-chain fatty acids (SCFAs). SCFAs can be absorbed by enterocyte and have many beneficial effects on host health. Neomycin-treated growing pigs showed a decrease in acetate in cecum (Gargallo and Zimmerman, 1980). Our previous study indicated that antibiotics reduced the concentrations of propionate and butyrate in feces of weaning piglets (Mu et al., 2017b). Lactate is a major fermentation product of carbohydrate metabolism, especially in the small intestine, which can reduce intestinal pH and inhibit the growth of pathogenic microbes (Mayeur et al., 2013). In addition to affecting the carbohydrate fermentation, antibiotics also affected profiles of metabolites involved in amino acid metabolism in the gut, such as biogenic amines, ammonia, and branched-chain fatty acid (BCFA) in the gut of growing or weaning piglets (Gargallo and Zimmerman, 1980; Bhandari et al., 2008; Mu et al., 2017b). Previous study demonstrated that neomycin increased ammonia concentration in the cecum of growing pigs (Gargallo and Zimmerman, 1980). Our previous study also showed that antibiotics significantly increased putrescine, cadaverine, and spermidine in the large intestine of weaning piglets (Mu et al., 2017b). These studies suggest marked effects of antibiotics in feed on microbial metabolism in weaning or growing pigs. Compared with weaning or growing pigs, the gut microbes of suckling piglets are less diverse and more dynamic. The early gut microbes’ colonization and associated metabolites at the suckling stage are important for the later microbial development, which further have impact on the growth and health of the pig (Arnal et al., 2014; Zhang et al., 2016). Thus, it is important to evaluate the impact of antibiotics exposure on gut microbial colonization and metabolism in suckling piglets.

Therefore, the current study was to investigate the effects of early life antibiotic exposure (from day 7 after birth to day 22) on gut microbial colonization and microbial metabolism in ileum and cecum of suckling piglets.

## Materials and Methods

### Ethics Statement

The animal experimental proposals were approved by the Animal Care and Use Committee of Nanjing Agricultural University and were in compliance with the Ethical Committee of Nanjing Agricultural University, Nanjing [authorization number SYXK (Su) 2011–0036].

### Animal, Diet and Experimental Design

This study was a part of series of studies designed to evaluate the effects of early life exposure of antibiotics on the gut microbes and nutrient metabolic of pigs and a detailed description of the experimental setup was reported in our previous studies (Mu et al., 2017b; Yu et al., 2017a). Briefly, 16 litters 7-day-old crossbred [Duroc × (Landrace × Large White)] suckling piglets were randomly assigned to one of the two groups (*n* = 8 litters/group): control group and antibiotics group. While sucking their sow’s milk from day 7 after birth to day 22, the piglets in the control group were fed a commercial creep feed (as shown in Supplementary Table S1) without any antibiotic and the antibiotic group piglets were fed the same commercial creep feed with a mixture of antibiotics (50 mg/kg olaquindox, 50 mg/kg oxytetracycline calcium, and 50 mg/kg kitasamycin) (Antibiotic). This mixture of antibiotics have broad spectrum of antibacterial activity as described previously (Yu et al., 2017a), and is commonly used as a growth promoter in creep feed for suckling and weaning piglets in commercial farms in China. The sow was fed a commercial corn-soybean based diet (as shown in Supplementary Table S2), which was produced by Cargill Investments (China) Ltd. (Shanghai, China) and contained no antibiotics. While sucking their respective sow’s milk, the piglets were fed twice per day (08:00 and 17:00 h, equal portions at each meal). At day 23, 8 piglets (*n* = 8 barrows) from each group was randomly selected (one piglet from each litter) and fasted for approximately 12 h before they were euthanized. The digesta in the ileum and cecum were collected and mixed, respectively, as described previously (Mu et al., 2017b). The samples were then stored at -80°C until further DNA extraction and metabolism analysis.

### Chemical Composition Analysis

Short-chain fatty acid concentrations in ileal and cecal digesta were measured by gas chromatography (GC) as described in our previous study (Yu et al., 2017b). Briefly, approximately 0.4 g of ileal and cecal digesta were mixed with 1.6 mL double distilled water. The mixture was vortexed and centrifuged at 13,000 × *g* for 10 min at 4°C. A portion of 1 mL supernatant was transferred to a new tube and mixed with 0.2 mL 25% (w/v) metaphosphoric acid. After homogenization, the samples were stored at -20°C for 12 h to precipitate the proteins. After thawing, the mixture was centrifuged at 13,000 × *g* for 10 min at 4°C. The supernatant was filtered through a 0.22-μm syringe filter and then analyzed on an Aglient 7890B system with a flame ionization detector (Agilent Technologies Inc., United States). Lactate concentration in ileal and cecal digesta was determined by the enzymatic colorimetric method with Olympus AU2700 auto analyzer (Olympus, Tokyo, Japan) and reagents were purchased from Nanjing Jiancheng Biological Engineering Institute (Nanjing, China).

For ammonia analysis, the ammonia concentration of ileum and cecum digesta were acidified with 0.2 mol/L HCl and analyzed using UV spectrophotometer according to Chaney and Marbach (1962). Amine concentrations in the ileum and cecum digesta were determined by high-performance liquid chromatography (HPLC) with precolumn dansylation as described in a previous study (Yang et al., 2014). Briefly, 0.5 g ileal and cecal digesta were weighted into a 2-mL centrifuge tube, 1.5 mL 5% (w/v) trichloroacetic acid solution was added to precipitate the proteins and peptides. After extraction by n-hexane, a portion of 0.5 mL subnatant was derived using dansyl chloride. The gradient elution of two mobile phase were used as follows: mobile phase A consisted of HPLC grade water and mobile phase B was acetonitrile. The flow rate was 1.0 mL/min. The wavelength of ultraviolet detector was 254 nm and the column temperature was 30°C.

### DNA Extraction and Preparation of Amplicons for High-Throughput Pyrosequencing

Total genomic DNA in the ileal and cecal digesta was extracted with the bead-beating and phenol-chloroform extraction methods as suggested by previous study (Zoetendal et al., 1998). The concentration of DNA was quantified using a NanoDrop spectrophotometer (Thermo Fisher Scientific Inc., Wilmington, DE, United States). The V3-V4 region of the bacterial 16S rRNA genes were amplified by PCR (Initial denaturation program at 95°C for 2 min, followed by 25 cycles at 95°C for 30 s, 55°C for 30 s, and 72°C for 30 s, and a final extension at 72°C for 5 min) using primers 338F (5′- ACT CCT RCG GGA GGC AGC AG-3′) and 806R (5′-GGA CTA CCV GGG TAT CTA AT-3′) (Mao et al., 2015). PCR products were visualized on a 2% (w/v) agarose gels and purified with AxyPrep DNA Gel Extraction Kit (Axygen Biosciences, Union City, CA, United States) according to the manufacturer’s instructions and quantified using QuantiFluor^TM^-ST (Promega, United States). Equal molar ratios of purified amplicons were pooled from each sample and paired-end sequenced (2 × 250) on an Illumina MiSeq platform according to standard protocols (Caporaso et al., 2012).

### Bioinformatics Analyses

The raw sequences from the Illumina MiSeq platform were demultiplexed and quality-filtered using the QIIME (version 1.7.0) software package (Campbell et al., 2010), as described by Mu et al. (2016). Operational taxonomic units (OTUs) were clustered with a cutoff of 97% similarity using UPARSE (version 7.1^1^), and chimeric sequences were identified and removed using UCHIME (Edgar, 2010). Representative sequences from each OTU were obtained and classified with a confidence level of 90% using 16S rRNA sequences from Silva release 119^2^ (Quast et al., 2013). Some representative sequences were also loaded into the National Center for Biotechnology Information Basic Local Alignment Search Tool website against the 16S rRNA sequence database (Altschul et al., 1990). Bacterial diversity was estimated with rarefaction analysis, an abundance-based coverage estimator (ACE), a bias-corrected Chao richness estimator, and the Shannon and Simpson diversity index using the MOTHUR program (version v.1.35.0^3^) (Schloss et al., 2009). The Bray–Curtis similarity clustering analysis was used to perform a principal coordinate analysis (PCoA) (Gonzalez-Silva et al., 2017), and a distance-based analysis of molecular variance (AMOVA) was conducted to assess the significant differences between antibiotic and control group samples (Schloss et al., 2009). Linear discriminant analysis effect size (LEfSe) analysis was performed to pick the significant and unique OTUs in each group using a linear discriminant algorithm (LDA) effect size (i.e., LDA score, > 2).

The 16S sequencing data in this paper were submitted to the GenBank Sequence Read Archive database under accession number SRP 132384.

### Statistical Analysis

Statistical analysis was carried out with tests using the SPSS software package (SPSS v. 20, SPSS Inc., Chicago, IL, United States). The normality of the distribution of variables was assessed with Shapiro–Wilk tests. The microbial metabolites, microbial diversity data, and data of taxa richness found to have a normal distribution were analyzed by the independent-samples *t*-test procedure. The Mann–Whitney test was used to analyze variables found to have a non-normal distribution (some data of taxa richness). False discovery rate (FDR) correction was also used to verify the discriminant bacterial community data (Benjamini and Hochberg, 2000). Data were expressed as the means ± SEM. Differences were considered significant at *P* ≤ 0.05, and tendency was declared with 0.05 < *P* < 0.10. Correlation between microbial fermentation metabolites with bacterial abundance (genus proportion from pyrosequencing analysis) were analyzed by Pearson’s correlation test using GraphPad Prism version 5.0 (GraphPad Software, San Diego, CA, United States). Correlation was considered significant when the absolute value of Pearson correlation coefficient was >0.5 and statistically significant (*P* < 0.05).

## Results

### Growth Performance

In this study, early antibiotics exposure did not affect the average daily gain compared with control group (330.15 ± 30.12 vs. 350.45 ± 20.56, *P* = 0.749). The average daily feed intake was also similar between the control group and antibiotics group (16.98 ± 0.75 vs. 17.29 ± 0.56, *P* = 0.709).

### Effects of Early Antibiotics Exposure on Ileal and Cecal Bacterial Community Structure Revealed by Pyrosequencing

**FIGURE 1:**
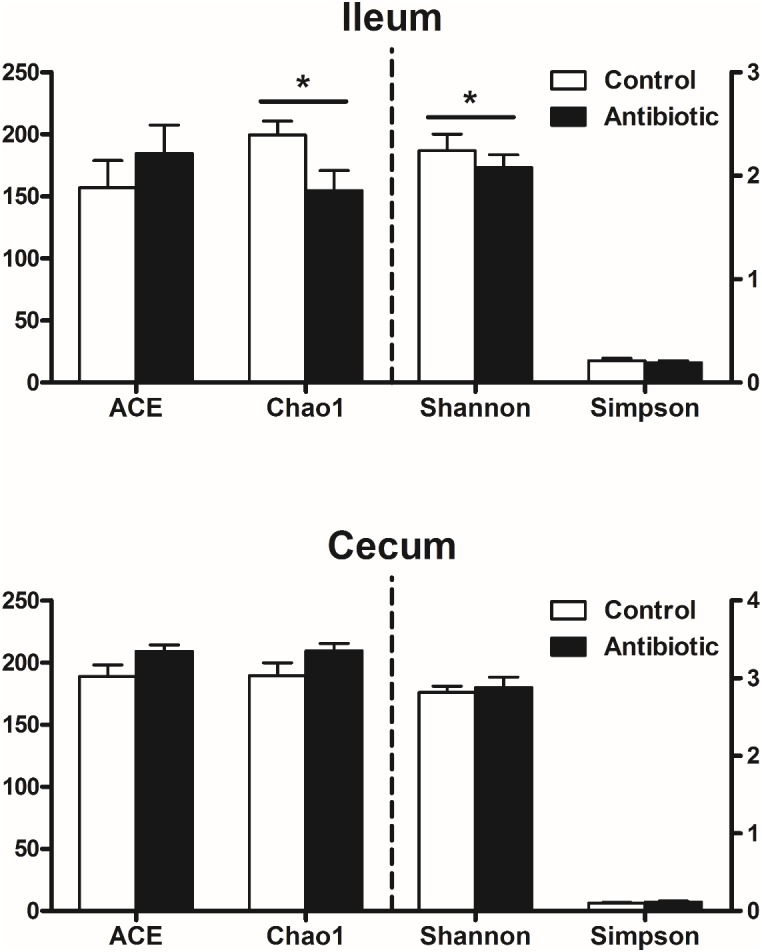
Effects of early antibiotics exposure on the diversity of ileal and cecal bacterial community at the 3% dissimilarity level. The values are expressed as the means ± SEM, with eight piglets per group. Asterisks indicated statistically significant difference from control: ^∗^*P* < 0.05, ^∗∗^*P* < 0.05.

**FIGURE 2:**
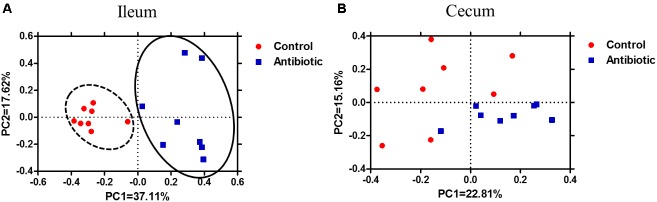
Principle coordinate analysis of **(A)** ileum samples and **(B)** cecum samples in the control and antibiotic group by Bray–Curtis similarity metric. The percentage of variation explained by PC1 and PC2 are indicated in the axis.

A total of 1,173,338 V3-V4 16S rRNA sequence reads from the 32 samples, with an average 36,666 sequence reads for each sample were used for subsequent analysis. As shown in Supplementary Figure S1, the rarefaction curves tended to approach a plateau, indicating that further sequencing would not result in an effective increase of OTUs in each group. The bacterial richness and diversity at a genetic distance of 3% in each sample are presented in **Figure 1**. In the ileum, the antibiotics decreased the species richness and diversity indices compared to that in the control group, as reflected by the Chao 1 and Shannon index with statistical differences. However, in the cecum, as compared with the control group, the antibiotics did not affect the species richness (ACE and Chao 1) and diversity indices (Simpson index and Shannon index). The Bray–Curtis similarity metric in MOTHUR was used to evaluate β-diversity across the sample (**Figure 2**). The PCoA result indicated a distinct separation in microbiota composition between the antibiotics group and the control in ileum (**Figure 2A**; axis 1 + axis 2 = 54.76%). AMOVA analysis, which evaluates the statistical significance of the spatial separation that was observed among the different groups in PCoA plots, indicated that the antibiotics significantly affected the ileum microbial communities (*F*s = 9.12, *P* < 0.001). However, cecal samples of the piglets from the antibiotics group and the control group were not separated (**Figure 2B**).

**FIGURE 3:**
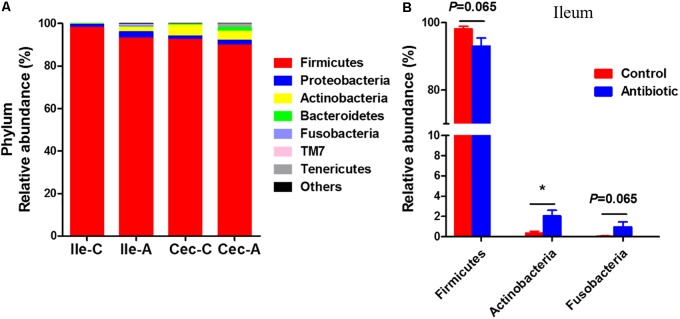
Phylum-level composition. **(A)** The phylum-level taxonomic composition of Average relative ileal and cecal microbiota in suckling piglets. **(B)** The changed bacterial phyla found in ileum. The values are expressed as the means ± SEM, with eight piglets per group. Asterisks indicated statistically significant difference from control (Mann–Whitney *U*-test): ^∗^*P* < 0.05 Abbreviations: Ile-C, Ileum-Control; Ile-A, Ileum-Antibiotic; Cec-C, Cecum-Control; Cec-A, Cecum-Antibiotic.

At phylum level, the Firmicutes was the most predominant phylum in the ileum and cecum of piglets (**Figure 3A**), accounting for more than 90% of total sequences. In the ileum (**Figure 3B**), the antibiotics tended to reduce the abundance of Firmicute*s* (*P* = 0.065). The antibiotics significantly increased the abundance of bacteria belonging to the phyla Actinobacteria (*P* < 0.05), and tended to increase the abundance of Fusobacteria (*P* = 0.065). However, no significant changes in the abundance of bacteria belonging to the phyla Bacteroidetes, Proteobacteria, TM7, and Tenericutes was observed between the control and antibiotic groups. In the cecum, none of the bacterial phyla changed in abundance after antibiotics exposure.

**FIGURE 4:**
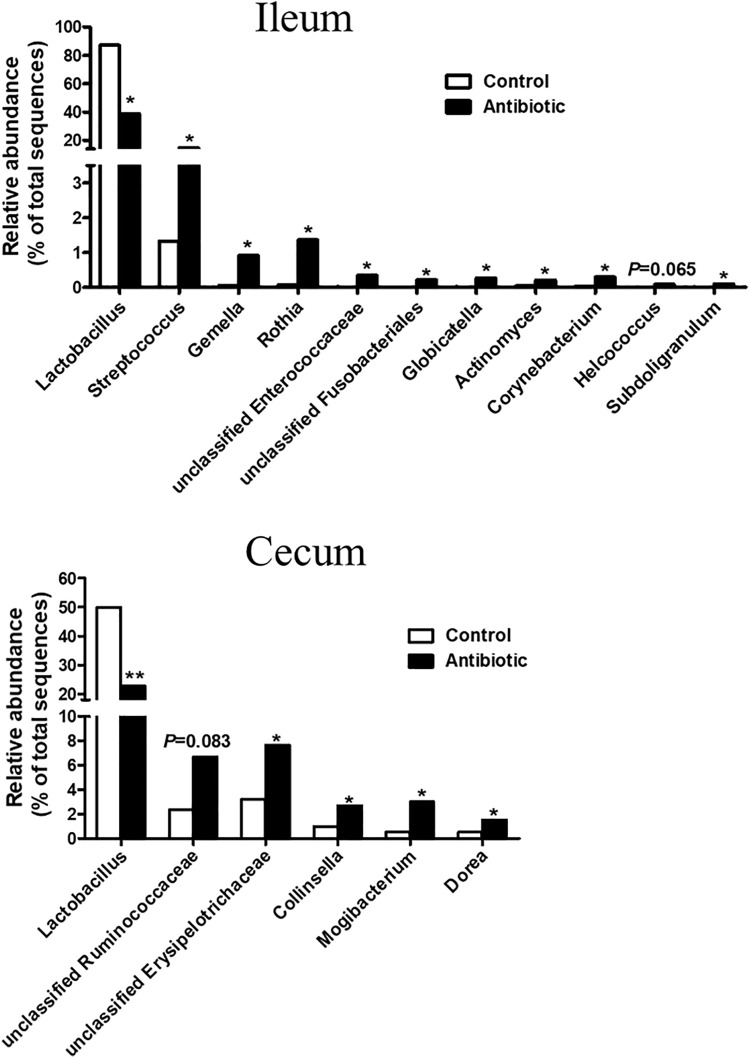
Effect of early antibiotics exposure on the significantly changed genera in ileal and cecal digesta. The values were expressed as the medians, with eight piglets per group. Asterisks indicated statistically significant difference from control (Mann–Whitney *U*-test): ^∗^*P* < 0.05, ^∗∗^*P* < 0.01.

The 30 most abundant genera are listed in Supplementary Figure S2 for ileum samples and Supplementary Figure S3 for cecum samples. In ileum samples, *Lactobacillus*, *Streptococcus*, and *Bacillus* were the abundant genera (>2% in at least one group) (Supplementary Figure S1). The antibiotics significantly reduced the abundance of *Lactobacillus*, increased the abundance of *Streptococcus*, *Rothia*, unclassified Enterococcaceae, unclassified Fusobacteriales, *Globicatella*, *Actinomyces*, *Corynebacterium*, and *Subdoligranulum* (**Figure 4**). Meanwhile, the antibiotics also tended to increase the abundance of *Gemella* and *Helcococcus* (*P* = 0.083, *P* = 0.065, respectively). In the cecum, *Lactobacillus*, *Streptococcus*, *Subdoligranulum*, unclassified Ruminococcaceae, unclassified Peptostreptococcaceae, and unclassified Erysipelotrichace were the abundant genera (>5% in at least one group) (Supplementary Figure S2). The antibiotics treatment significantly decreased the abundance of *Lactobacillus* (*P* < 0.05), increased the abundance of unclassified Erysipelotrichace, *Collinsella*, *Mogibacterium*, and *Dorea*, and tended to increase (*P* < 0.05) the abundance of unclassified Ruminococcaceae (*P* = 0.083) (**Figure 4**).

**FIGURE 5:**
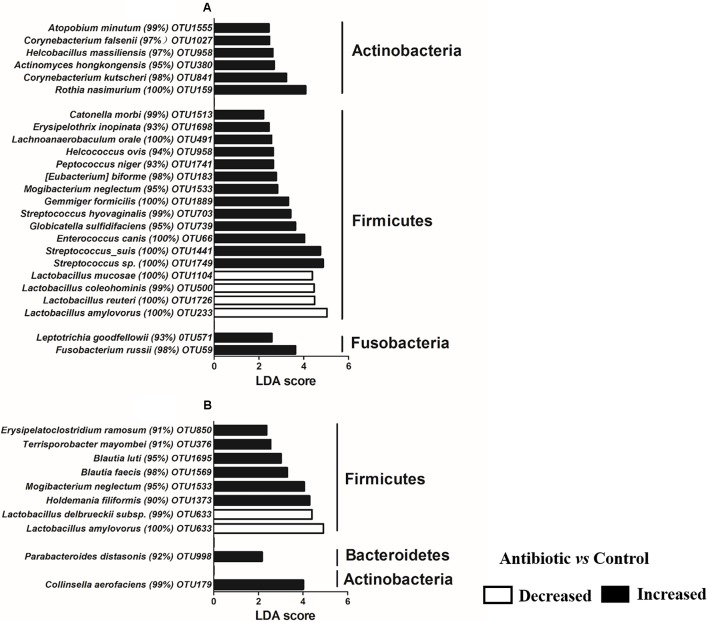
Significantly changed bacteria OTUs in ileum **(A)** and cecum **(B)** by antibiotic treatment at OTUs levels, as revealed by LefSe analysis. An LDA score of >2 was considered significant.

To identify specific bacteria that are characteristic for antibiotics treatment, LEfSe analysis was performed at the OTUs level (**Figure 5**). In the ileum, 25 OTUs were significantly different between the antibiotics and the control (**Figure 5A**). Among the different OTUs, 21 of these OTUs were higher in the antibiotic treatment group and 4 OTUs were higher in the control group (*P* < 0.05). In the cecum, 10 OTUs were different (**Figure 5B**). Eight of these OTUs were higher in antibiotic treatment group, while 2 OTUs were higher in control group (*P* < 0.05). Taken together, these results indicate that the early antibiotics exposure significantly changed the intestinal microbiota, especially in the ileum digesta.

### Effects of Early Antibiotics Exposure on Fermentation Metabolites in Ileum and Cecum Digesta

**FIGURE 6:**
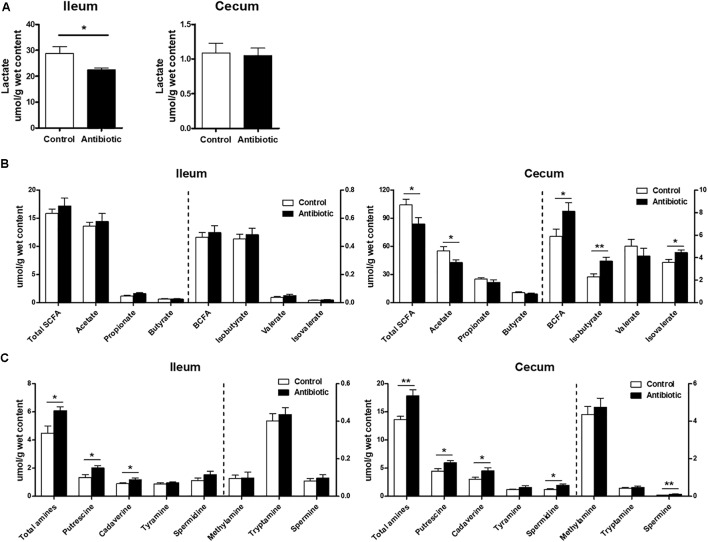
Effect of early antibiotics exposure on the microbial metabolites in ileum and cecum content of piglets. **(A)** Lactate; **(B)** SCFAs; **(C)** Amines. The values are expressed as the means ± SEM, with eight piglets per group. Asterisks indicated statistically significant difference from control (Student’s *t*-test: ^∗^*P* < 0.05, ^∗∗^*P* < 0.01).

Short-chain fatty acids are the major microbial products from carbohydrates fermentation in the gut, especially in the large intestine. As shown in **Figure 6B**, the cecum had large amount of SCFAs and the antibiotic decreased (*P* < 0.05) the concentrations of acetate and total SCFA compared to those in control group. Meanwhile, the antibiotic group also affected the minor SCFAs, with the concentrations 37.6, 20.2, and 27.7% higher (*P* < 0.05) for isobutyrate, isovalerate and BCFA, respectively, than those in the control group. In the ileum (**Figure 6B**), the amount of SCFA was low and the antibiotics showed no effect on the level of SCFA (*P* > 0.05). Lactate is a major product in the intestine especially in the small intestine from carbohydrate fermentation by lactic acid-producing bacteria, such as *Lactobacillus,* *Bifidobacterium*, and *Enterococcus*. High level of lactate in the small intestine is important to prevent the proliferation of pathogenic or harmful bacteria and maintain the intestinal health. As shown in **Figure 6A**, the concentration of lactate in the ileal digesta was high and the antibiotics significantly decreased the concentration of lactate (*P* < 0.05). The cecum had little lactate and the antibiotics had no effect in cecum (*P* > 0.05).

Ammonia and biogenic amines are derived from deamination and decarboxylation of amino acids, respectively. For ammonia, the cecum showed higher level of ammonia than the ileum and the antibiotics did not affect the level of ammonia either in the ileum or in the cecum (data not shown). For biogenic amines (**Figure 6C**), putrescine and putrescine were the two main amines both in the ileum and cecum, but the amount of the amines in the cecum was high, while that in the ileum was little and some were negligible. The antibiotics significantly increased (*P* < 0.05) the concentrations of putrescine, cadaverine, and total amines in the ileum. In the cecum, the antibiotics treatment also increased the concentrations of putrescine, cadaverine, spermidine, spermine, and total amines, but did not affect the concentrations of methylamine, tryptamine, and tyramine. Collectively, these results indicated that the antibiotics markedly increased the concentrations of amines in the ileum and cecum, suggesting a great impact of antibiotics on amino acid metabolism in the intestine.

### Correlation Between Bacterial Fermentation Productions With Bacteria

**FIGURE 7:**
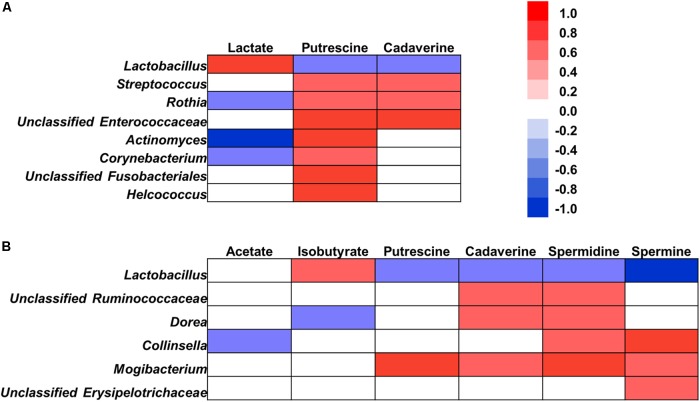
Correlation analysis between the abundance of microbiota (at the genus level) and microbial metabolites in the ileum **(A)** and cecum **(B)**. Correlation was considered significant when the absolute value of Pearson correlation coefficient was >0.5 and statistically significant (*P* < 0.05). The red represents a significant positive correlation (*P* < 0.05), blue represents a significant negative correlation (*P* < 0.05), and white represents a non-significant correlation (*P* > 0.05).

To understand the relationships between the microbial composition changes and the change in microbial fermentation profiles during the antibiotic exposure, a Pearson’s correlation analysis was performed between microbial composition and metabolites concentrations (**Figure 7**). In the ileum (**Figure 7A**), there were significantly correlations between the changes of lactate concentration and bacteria, with positive correlation with the abundance of *Lactobacillus*, while negative correlation with the abundance of *Rothia*, *Actinomyces*, *Corynebacteriales*. With amines, the concentrations of putrescine and cadaverine negatively correlated with the abundance of *Lactobacillus*, while positively correlated with the abundance of *Streptococcus*, *Rothia*, and unclassified Enterococcaceae. Putrescine also positively correlated with the abundance of *Actinomyces*, *Corynebacteriales*, unclassified Fusobacteriales and *Helcococcus*. In the cecum (**Figure 7B**), the concentration of acetate showed negative correlations with the abundance of *Collinsella*. Isobutyrate concentration was positively correlated with the abundance of *Lactobacillus*, while negatively correlated with the abundance of *Dorea*. With amines, concentrations of putrescine, cadaverine, spermidine, and spermine correlated negatively with the abundance of *Lactobacillus*, and correlated positively with the abundance of *Mogibacterium*. Cadaverine and spermidine concentrations were also positively correlated with the abundance of unclassified Ruminococcaceae and *Dorea*. In addition, spermidine and spermine concentrations were positively correlated with the abundance of *Collinsella*, and spermine concentration also positively correlated with the abundance of unclassified Erysipelotrichaceae. In general, these results indicate that the changes in intestinal microbiota are correlated with alterations of metabolites.

## Discussion

Antibiotics have been widely used as growth promoters in swine husbandry for many years. The effect of early life antibiotics exposure on the microbiota in the weaning piglets or growing pigs have been widely described. In the present study, we investigated the response of the ileal and cecal digesta of microbes of the suckling piglets to antibiotic treatment using high-throughput sequencing. The antibiotics markedly affected the ileal microbiota, with a sharp reduction in the abundance of *Lactobacillus*, and dramatically decreased the lactate concentration in the ileum and SCFA in the cecum, while increased the concentrations of protein fermentation products (BCFA, putrescine, cadaverine, spermidine, and spermine) in the ileum and cecum. These findings indicate a marked impact of the antibiotics on the intestinal microbial community and metabolic profiles of the suckling piglets.

### Early Antibiotics Exposure Altered the Microbial Community Structure of the Suckling Piglets, With More Evident Effect in the Ileum Than in the Cecum

In the present study, antibiotics had varying impact on the microbial composition in the ileum and cecum, with dramatic effect in the ileum and less effect in the cecum, as revealed by the diversity and richness of microbiota, and the numbers and types of genera affected. With weaning piglets, our previous study also observed antibiotics-induced location-specific shift in the gut microbiota, with dramatic impact in the stomach and small intestine, but had less impact in the hindgut (Mu et al., 2017a). Different locations in the lumen may differ in physicochemical and nutrient conditions for different microbial communities (Pereira and Berry, 2017). Therefore, the different microbial distribution along the gut may account for the different alterations in the ileal and cecal microbiota after antibiotic treatment.

Diversity and richness index are often used as an indicator of functional resilience of microbial ecosystems in the gut. In the present study with suckling piglets, the antibiotics exposure decreased the diversity (Shannon) and richness index (Chao 1) of microbiota in the ileum, which was confirmed by analysis of Bray–Curtis PCoA (**Figure 2A**) and AMOVA. Additionally, antibiotics exposure increased the abundance of Actinobacteria and affected 10 genera or similar groups. However, in weaning piglets, antibiotics exposure did not affect the diversity and richness of microbiota in the ileum though with evident effect in the stomach and jejunum, and had no effect on the abundance of Actinobacteria and did not affect abundances of any genera (Mu et al., 2017a). The difference between piglets in the ileum in response to the antibiotics may primarily be due to the age of the piglets as the gut microbiota of suckling piglets (relatively younger age, at day 23) is less diverse but more dynamic than weaning piglets (relatively older age). Our previous study, investigating succession of gut microbiota of the piglets from the immediate birth until 3 weeks after weaning, showed an age-dependent manner of the gut microbiota development and acquisition (Bian et al., 2016). Thus, the less diverse and more dynamic microbial community in the gut of the suckling piglets may be more vulnerable than the weaning pigs and thus can be easily influenced by environmental factors such as the antibiotic exposure in the present study.

With specific genera, the antibiotics markedly reduced the genus *Lacobacillus* in the ileum, while increased many genera, such as *Streptococcus*, *Rothia*, and *Corynebacterium*. The genus *Lactobacillus* is the most predominant genus in the small intestine. However, with weaning piglets at day 42, the same antibiotics did not show significant effect on the *Lactobacillus* (Mu et al., 2017a). This different response may be due to the different relative abundance of the *Lactobacillus* in the intestine as the event of weaning could change the relative abundances of bacteria groups (Bian et al., 2016). Our previsous study showed that the abundance of *Lactobacillus* quantified by real-time PCR changed dramatically from before weaning to after weaning (9.29 vs. 6.80 16S rRNA gene copies/g wet weight digesta) (Su et al., 2008). In the present study with suckling piglets, *Lactobacillus* is the most predominant genus (87.05% vs. 38.93% of total sequence in the control and antibiotic group, respectively) in the ileum, greater than the *Strepotococcus* (2.30% vs. 16.01% of total sequence in the control and antibiotic group, respectively). With weaning piglets at 42 days of age, the most predominant genus is *Strepotococcus* (17.56% vs. 10.61% of total sequence in the control and antibiotic group, respectively), and the *Lactobacillus* (10.42% vs. 11.32% of total sequence in the control and antibiotic group, respectively) was the second predominant genus. In a rat model, previous study indicated that antibiotic treatment (amoxicillin) from postnatal day 7 until days 17 or 21, nearly eradicated *Lactobacillus* in the small intestine and colon (Schumann et al., 2005). In chickens, antibiotic treatment from postnatal days 1–7 also significantly decreased the abundance of some species within the genera *Lactobacillus* (Simon et al., 2016). These results suggest that the most predominant genus *Lactobacillus* in the neonates is very sensitive to antibiotics. *Lactobacillus* bacteria are known as potentially beneficial species in the gut, which can prevent the infection or colonization of pathogens by competition for epithelial binding sites and nutrients, and produce some antimicrobial factors, such as bacteriocins and lactic acid (Hamnes et al., 1992). Thus, the use of antibiotics either for improving growth performance or treatment of diseases could also reduce the abundance of potentially benefical species, which could make the intestine rather vulnerable.

On the countrary, the antibiotics exposure increased the abuncance of many other genera, such as *Streptococcus*. Some species within the genera *Streptococcus*, including *S. suis* (Lun et al., 2007) and *S. gallolyticus* (Boleij and Tjalsma, 2013), are known as opportunistic pathogens that induce mortality of piglets after weaning. With suckling piglets in the present study, the antibiotics significantly increased the abundance of *S. suis* and *S. gallolyticus* (**Figure 5A**). However, with growing pigs, previous study showed that in-feed antibiotics (chlortetracycline, sulfamethazine, and penicillin) treatment decreased the abundance of *Streptococcus* in the ileum and cecum of growing pig (Looft et al., 2014). In contrast to the established and stable microbiota of growing pigs, the gut microbiota of suckling piglets is not mature and may be more vulnerable to disturbance such as antibiotics exposure.

Collectively, our results suggested that early antibiotics exposure altered the microbial community structure of suckling piglets toward a vulnerable gut environment, and with more evident effect in the ileum than in the cecum. For the suckling piglets, the gut microbiota is dynamic and vulnerable to pathogen invasion, and the early colonization of gut microbiota has important role in the health of the host. Thus, based on the results from suckling piglets in the present study, beneficial microbes or probiotics could be used instead of antibiotics in the feed of animals.

### Early Antibiotics Exposure Markedly Reduced Lactate Concentration in the Ileum of the Suckling Piglets

Lactate is an important bacterial fermentation product of carbohydrate metabolism in the gut especially in the small intestine, which can reduce gastrointestinal tract pH, inhibit the multiplication of enterotoxigenic *Escherichia coli*, reduce the mortality rate of the animals, and is more effective than other organic acids in improving pig growth performance (Tsiloyiannis et al., 2001; Suiryanrayna and Ramana, 2015). The level of lactate in the intestine is the result of three processes occurring simultaneously: lactate production by lactate-producing bacteria, lactate consumption by lactate-utilizing bacteria, and intestinal absorption (Pham et al., 2016). *Lactobacillus* is considered as the main lactate producer in the intestine, and in the present study was positively correlated with the lactate concentration in the ileum (**Figure 7A**). Thus, many species or strains belonging to *Lactobacillus* have been used as probiotics in piglets (Lallès et al., 2007). Some bacteria within *Actinomyces* can convert lactate into acetate and CO_2_ (Takahashi and Yamada, 1999), and *Rothia* strains utilize L-lactate to produce pyruvate and hydrogen peroxide (H_2_O_2_) (Lim et al., 2013). The present study also indicated that antibiotic significantly increased the abundance of *Actinomyces* and *Rothia* (**Figure 4**), and negatively correlated with the concentration of lactate in the ileum (**Figure 7A**). Thus, the reduced abundance of lactate-producing bacteria and increased abundance of lactate-utilizing bacteria contributed to the reduction of ileal lactate after antibiotic exposure. Taken together, the decrease of lactate concentration after antibiotics exposure suggests that early antibiotics exposure may make the small intestine more vulnerable to some pathogenic bacteria.

### Early Antibiotics Exposure Markedly Reduced SCFAs Concentrations in the Cecum of the Suckling Piglets

Short-chain fatty acids are the main microbial fermentation products in the gut especially in the large intestine. The cecum is the major site of microbial fermentation of undigested carbohydrate or protein in pigs, as the cecum had a greater richness of bacteria (10^10^–10^12^) and a much longer retention time of digesta (20–38 h) when compared with the ileum (10^5^–10^9^, 2–6 h, respectively; Low and Zebrowska, 1989). In the present study, antibiotics exposure markedly reduced SCFAs concentrations in the cecum. SCFA can be absorbed by enterocyte. It has been reported that orally administrated antibiotics affected the permeability of small intestinal enterocytes and consequently may affect nutrient absorption. However, until now, there was no direct evidence that orally administrated antibiotics could affect the absorption of SCFA by enterocyte of the large intestine. Our recent study using transcriptomics of intestine tissues indicated that the same antibiotics treatment altered the expression of genes related to metabolic processes in the jejunum, but not in ileum of piglets at 42 days (Yu K. F. et al., 2017). Thus, the orally administrated antibiotics in this study may have little effect on the SCFA absorption in the large intestine. On the other hand, antibiotics-induced alteration of microbial composition can change SCFA profile in the colon and feces (Mu et al., 2017b), in consistent with the decrease in cecum in the present study. It is possible that the antibiotic effect on the nutrient absorption in the small intestine could change the nutrient flow to the large intestine, and subsequently affect the microbiota in the large intestine, leading to an alteration of SCFA profile. Therefore, the decrease of SCFA in the cecum in the present study may be mainly due to the alteration of microbial composition after the oral administration of antibiotics. SCFA have many beneficial effects on host health. Some inflammatory bowel disease patients had lower levels of SCFAs in the feces compared to healthy individuals (Huda-Faujan et al., 2010), suggesting an association between low level of SCFA and unhealthy gut. Thus, the decrease of SCFA concentration in the present study may suggest an unhealthy gut environment after the antibiotics exposure.

However, the antibiotics exposure significantly increased the concentrations of isobutyrate and isovalerate, the BCFA (**Figure 6B**). Isobutyrate and isovalerate are only derived from the deamination of valine and leucine, respectively, which are often considered as indicators of amino acids catabolism in the gut (Blachier et al., 2007). The increase of cecal BCFA in the present study may suggest a promotion of microbial protein fermentation in the gut of suckling piglets after antibiotics exposure, which may be not beneficial to the gut health.

### Early Antibiotics Exposure Markedly Increased Amines Concentrations in the Ileum and Cecum of the Suckling Piglets

The antibiotics exposure markedly changes of amines profile in the ileum and cecum (**Figure 6C**). Among the amines, putrescine and cadaverine are the main amines, which are formed from decarboxylation of ornithine and arginine, lysine, respectively (Davila et al., 2013). The consistent increase of putrescine and cadaverine concentrations in the ileum and cecum after the antibiotics in the present study indicated an increased precursor amino acid decarboxylation. Meanwhile, the increase of putrescine-derived spermine and spermidine concentration in the cecum further suggested an increased decarboxylation by the bacteria in the gut. Our previous research also demonstrated an antibiotics-induced increase in concentration of putrescine and cadaverine in the cecum of weaning piglets (Mu et al., 2017b). Amines may exert varying physiological effects on the host. For example, some amines, such as tryptamine can stimulate the secretion of serotonin by enterochromaffin cells and regulate intestinal motility (Yano et al., 2015). Spermidine and spermine have been shown to be essential for somatic cell growth (Davila et al., 2013). On the other hand, high concentrations of amines such as putrescine and cadaverine can exert adverse impact on the host, inducing oxidative stress and DNA damage, and then increase the tumorigenesis risk (Holmes et al., 2011), and can also increase resistance of human pathogens (*Neisseria gonorrhoeae*) to mediators of innate immune defense (Goytia and Shafer, 2010). Additionally, above certain threshold levels in the gut, amines may produce detrimental effects such as increasing the incidence of diarrhea (Rist et al., 2013). Although whether the increase of potentially harmful amines after antibiotic exposure affects the gut health in suckling piglets still needs further research, our findings suggest that the early antibiotics exposure altered gut microbial composition and the function of this community likely toward an unhealthy gut environment.

## Conclusion

This study, using high-throughput sequencing the Miseq platform and bioinformatics analyses, demonstrated that early life antibiotics exposure altered the ileal and cecal microbial composition and metabolic profiles of the suckling piglets, likely toward an unhealthy gut environment by increasing the abundance of opportunistic pathogens while reducing the abundance of *Lactobacillus*. The antibiotics exposure markedly reduced lactate concentration in the ileum and SCFAs in the cecum, while increased the concentrations of microbial metabolites derived from amino acid decarboxylation (BCFA, putrescine, cadaverine, spermidine, and spermine). These alterations may help us to understand the negative effects of early antibiotics exposure on gut microbial composition and metabolism of animals and humans. The findings provide evidence that antibiotics use for growth enhancement of livestock also brings additional gut microbiome health risks and implications.

## Author Contributions

WZ and YS conceived and designed the whole trial. CM and YY conducted the piglet trial. MY and CZ conducted the laboratory analyses. MY, CM, and WZ wrote the manuscript. All authors agreed to be accountable for all aspects of the work.

## Conflict of Interest Statement

The authors declare that the research was conducted in the absence of any commercial or financial relationships that could be construed as a potential conflict of interest.

## Footnotes

1 http://drive5.com/uparse/

2 http://www.arb-silva.de

3 http://www.mothur.org

## References

[B1] Allen H. K. Looft T. Bayles D. O. Humphrey S. Levine U. Y. Alt D. (2011). Antibiotics in feed induce prophages in swine fecal microbiomes. *MBio* 2:e00260-11. 10.1128/mBio.00260-11 22128350 PMC3225969

[B2] Altschul S. F. Gish W. Miller W. Myers E. W. Lipman D. J. (1990). Basic local alignment search tool. *J. Mol. Biol.* 215 403–410. 10.1016/S0022-2836(05)80360-2 2231712

[B3] Arnal M.-E. Zhang J. Messori S. Bosi P. Smidt H. Lallès J.-P. (2014). Early changes in microbial colonization selectively modulate intestinal enzymes, but not inducible heat shock proteins in young adult swine. *PLoS One* 9:e87967. 10.1371/journal.pone.0087967 24505340 PMC3913709

[B4] Benjamini Y. Hochberg Y. (2000). On the adaptive control of the false discovery rate in multiple testing with independent statistics. *J. Educ. Behav. Stat.* 25 60–83. 10.3102/10769986025001060 14584715

[B5] Bhandari S. Xu B. Nyachoti C. Giesting D. Krause D. (2008). Evaluation of alternatives to antibiotics using an *Escherichia coli* K88+ model of piglet diarrhea: effects on gut microbial ecology. *J. Anim. Sci.* 86 836–847. 10.2527/jas.2006-822 18192551

[B6] Bian G. R. Ma S. Q. Zhu Z. G. Su Y. Zoetendal E. G. Mackie R. (2016). Age, introduction of solid feed and weaning are more important determinants of gut bacterial succession in piglets than breed and nursing mother as revealed by a reciprocal cross-fostering model. *Environ. Microbiol.* 18 1566–1577. 10.1111/1462-2920.13272 26940746

[B7] Blachier F. Mariotti F. Huneau J.-F. Tomé D. (2007). Effects of amino acid-derived luminal metabolites on the colonic epithelium and physiopathological consequences. *Amino Acids* 33 547–562. 10.1007/s00726-006-0477-9 17146590

[B8] Boleij A. Tjalsma H. (2013). The itinerary of Streptococcus gallolyticus infection in patients with colonic malignant disease. *Lancet Infect. Dis.* 13 719–724. 10.1016/S1473-3099(13)70107-5 23831427

[B9] Campbell B. J. Polson S. W. Hanson T. E. Mack M. C. Schuur E. A. G. (2010). The effect of nutrient deposition on bacterial communities in Arctic tundra soil. *Environ. Microbiol.* 12 1842–1854. 10.1111/j.1462-2920.2010.02189.x 20236166

[B10] Caporaso J. G. Lauber C. L. Walters W. A. Berglyons D. Huntley J. Fierer N. (2012). Ultra-high-throughput microbial community analysis on the Illumina HiSeq and MiSeq platforms. *ISME J.* 6 1621–1624. 10.1038/ismej.2012.8 22402401 PMC3400413

[B11] Chaney A. L. Marbach E. P. (1962). Modified reagents for determination of urea and ammonia. *Clin. Chem.* 8 130–132. 13878063

[B12] Cho I. Yamanishi S. Cox L. Methé B. A. Zavadil J. Li K. (2012). Antibiotics in early life alter the murine colonic microbiome and adiposity. *Nature* 488 621–626. 10.1038/nature11400 22914093 PMC3553221

[B13] Conroy M. E. Shi H. N. Walker W. A. (2009). The long-term health effects of neonatal microbial flora. *Curr. Opin. Allergy Clin. Immunol.* 9 197–201. 10.1097/ACI.0b013e32832b3f1d 19398905

[B14] Davila A.-M. Blachier F. Gotteland M. Andriamihaja M. Benetti P.-H. Sanz Y. (2013). Intestinal luminal nitrogen metabolism: role of the gut microbiota and consequences for the host. *Pharmacol. Res.* 68 95–107. 10.1016/j.phrs.2013.01.003 23183532

[B15] Edgar R. C. (2010). Search and clustering orders of magnitude faster than BLAST. *Bioinformatics* 26 2460–2461. 10.1093/bioinformatics/btq461 20709691

[B16] Gargallo J. Zimmerman D. R. (1980). Effects of dietary cellulose and neomycin on function of the cecum of pigs. *J. Anim. Sci.* 51 121–126. 10.2527/jas1980.511121x 7410265

[B17] Gonzalez-Silva B. M. Rønning A. J. Andreassen I. K. Bakke I. Cervantes F. J. Østgaard K. (2017). Changes in the microbial community of an anammox consortium during adaptation to marine conditions revealed by 454 pyrosequencing. *Appl. Microbiol. Biotechnol.* 101 5149–5162. 10.1007/s00253-017-8160-5 28280868

[B18] Goytia M. Shafer W. M. (2010). Polyamines can increase resistance of *Neisseria gonorrhoeae* to mediators of the innate human host defense. *Infect. Immun.* 78 3187–3195. 10.1128/IAI.01301-09 20439477 PMC2897401

[B19] Hamnes W. Weiss N. Holzapfel W. (1992). “The genera *Lactobacillus* and *Carnobacterium*,” in *The Prokaryotes A Handbook on the Biology of Bacteria: Ecophysiology and Isolation, Identification, Applications* Vol. 2 eds Balows A. Truper H. G. Dworkin M. Harder W. Schleifer K.-H. (New York, NY: Springer-Verlag), 1536–1594.

[B20] Hansen C. H. F. Nielsen D. S. Kverka M. Zakostelska Z. Klimesova K. Hudcovic T. (2013). Patterns of early gut colonization shape future immune responses of the host. *PLoS One* 7:e34043. 10.1371/journal.pone.0034043 22479515 PMC3313961

[B21] Holmes E. Li J. V. Athanasiou T. Ashrafian H. Nicholson J. K. (2011). Understanding the role of gut microbiome–host metabolic signal disruption in health and disease. *Trends Microbiol.* 19 349–359. 10.1016/j.tim.2011.05.006 21684749

[B22] Hooper L. V. Littman D. R. Macpherson A. J. (2012). Interactions between the microbiota and the immune system. *Science* 336 1268–1273. 10.1126/science.1223490 22674334 PMC4420145

[B23] Huda-Faujan N. Abdulamir A. S. Fatimah A. B. Anas O. M. Shuhaimi M. Yazid A. M. (2010). The impact of the level of the intestinal short chain fatty acids in inflammatory bowel disease patients versus healthy subjects. *Open Biochem. J.* 4 53–58. 10.2174/1874091X01004010053 20563285 PMC2887640

[B24] Konstantinov S. R. Awati A. A. Williams B. A. Miller B. G. Jones P. Stokes C. R. (2006). Post-natal development of the porcine microbiota composition and activities. *Environ. Microbiol.* 8 1191–1199. 10.1111/j.1462-2920.2006.01009.x 16817927

[B25] Lallès J. P. Bosi P. Smidt H. Stokes C. R. (2007). Nutritional management of gut health in pigs around weaning. *Proc. Nutr. Soc.* 66 260–268. 10.1017/s0029665107005484 17466106

[B26] Lim Y. W. Schmieder R. Haynes M. Furlan M. Matthews T. D. Whiteson K. (2013). Mechanistic model of Rothia mucilaginosa adaptation toward persistence in the CF lung, based on a genome reconstructed from metagenomic data. *PLoS One* 8:e64285. 10.1371/journal.pone.0064285 23737977 PMC3667864

[B27] Looft T. Allen H. K. Cantarel B. L. Levine U. Y. Bayles D. O. Alt D. P. (2014). Bacteria, phages and pigs: the effects of in-feed antibiotics on the microbiome at different gut locations. *ISME J.* 8 1566–1576. 10.1038/ismej.2014.12 24522263 PMC4817603

[B28] Looft T. Johnson T. A. Allen H. K. Bayles D. O. Alt D. P. Stedtfeld R. D. (2012). In-feed antibiotic effects on the swine intestinal microbiome. *Proc. Natl. Acad. Sci. U.S.A.* 109 1691–1696. 10.1073/pnas.1120238109 22307632 PMC3277147

[B29] Low A. G. Zebrowska T. (1989). “Digestion in pig”, in *Protein Metabolism in Farm Animals Evaluation, Digestion, Absorption and Metabolism*, eds H.-D. Bock, B. O. Eggum, A. G. Low, O. Simon, and T. Zebrowska (Oxford: Oxford University Press), 53.

[B30] Lun Z. R. Wang Q. P. Chen X. G. Li A. X. Zhu X. Q. (2007). *Streptococcus suis*: an emerging zoonotic pathogen. *Lancet Infect. Dis.* 7 201–209. 10.1016/S1473-3099(07)70001-4 17317601

[B31] Mao S. Y. Zhang M. L. Liu J. H. Zhu W. Y. (2015). Characterising the bacterial microbiota across the gastrointestinal tracts of dairy cattle: membership and potential function. *Sci. Rep.* 5:16116. 10.1038/srep16116 26527325 PMC4630781

[B32] Mayeur C. Gratadoux J.-J. Bridonneau C. Chegdani F. Larroque B. Kapel N. (2013). Faecal D/L lactate ratio is a metabolic signature of microbiota imbalance in patients with short bowel syndrome. *PLoS One* 8:e54335. 10.1371/journal.pone.0054335 23372709 PMC3553129

[B33] Mu C. L. Yang Y. X. Luo Z. Guan L. L. Zhu W. Y. (2016). The colonic microbiome and epithelial transcriptome are altered in rats fed a high-protein diet compared with a normal-protein diet. *J. Nutr.* 146 474–483. 10.3945/jn.115.223990 26843585

[B34] Mu C. L. Yang Y. X. Su Y. Zoetendal E. G. Zhu W. Y. (2017a). Differences in microbiota membership along the gastrointestinal tract of piglets and their differential alterations following an early-life antibiotic intervention. *Front. Microbiol.* 8:797. 10.3389/fmicb.2017.00797 28536561 PMC5422473

[B35] Mu C. L. Yang Y. X. Yu K. F. Yu M. Zhang C. J. Su Y. (2017b). Alteration of metabolomic markers of amino-acid metabolism in piglets with in-feed antibiotics. *Amino Acids* 49 771–781. 10.1007/s00726-017-2379-4 28101652

[B36] Nicholson J. K. Holmes E. Wilson I. D. (2005). Gut microorganisms, mammalian metabolism and personalized health care. *Nat. Rev. Microbiol.* 3 431–438. 10.1038/nrmicro1152 15821725

[B37] Pereira F. C. Berry D. (2017). Microbial nutrient niches in the gut. *Environ. Microbiol.* 19 1366–1378. 10.1111/1462-2920.13659 28035742 PMC5412925

[B38] Pham V. T. Lacroix C. Braegger C. P. Chassard C. (2016). Early colonization of functional groups of microbes in the infant gut. *Environ. Microbiol.* 18 2246–2258. 10.1111/1462-2920.13316 27059115

[B39] Quast C. Pruesse E. Yilmaz P. Gerken J. Schweer T. Yarza P. (2013). The SILVA ribosomal RNA gene database project: improved data processing and web-based tools. *Nucleic Acids Res.* 41 590–596. 10.1093/nar/gks1219 23193283 PMC3531112

[B40] Rist V. Weiss E. Eklund M. Mosenthin R. (2013). Impact of dietary protein on microbiota composition and activity in the gastrointestinal tract of piglets in relation to gut health: a review. *Animal* 7 1067–1078. 10.1017/s1751731113000062 23410993

[B41] Romick-Rosendale L. E. Goodpaster A. M. Hanwright P. J. Patel N. B. Wheeler E. T. Chona D. L. (2009). NMR-based metabonomics analysis of mouse urine and fecal extracts following oral treatment with the broad-spectrum antibiotic enrofloxacin (Baytril). *Magn. Reson. Chem.* 47(Suppl. 1), S36–S46. 10.1002/mrc.2511 19768747

[B42] Saavedra J. M. Dattilo A. M. (2012). Early development of intestinal microbiota: implications for future health. *Gastroenterol. Clin. North Am.* 41 717–731. 10.1016/j.gtc.2012.08.001 23101683

[B43] Schloss P. D. Westcott S. L. Ryabin T. Hall J. R. Hartmann M. Hollister E. B. (2009). Introducing mothur: open-source, platform-independent, community-supported software for describing and comparing microbial communities. *Appl. Environ. Microbiol.* 75 7537–7541. 10.1128/AEM.01541-09 19801464 PMC2786419

[B44] Schokker D. Zhang J. Zhang L. L. Vastenhouw S. A. Heilig H. G. Smidt H. (2014). Early-life environmental variation affects intestinal microbiota and immune development in new-born piglets. *PLoS One* 9:e100040. 10.1371/journal.pone.0100040 24941112 PMC4062469

[B45] Schumann A. Nutten S. Donnicola D. Comelli E. M. Mansourian R. Cherbut C. (2005). Neonatal antibiotic treatment alters gastrointestinal tract developmental gene expression and intestinal barrier transcriptome. *Physiol. Genomics* 23 235–245. 10.1152/physiolgenomics.00057.2005 16131529

[B46] Simon K. Verwoolde M. B. Zhang J. Smidt H. Reilingh G. D. V. Kemp B. (2016). Long-term effects of early life microbiota disturbance on adaptive immunity in laying hens. *Poult. Sci.* 95 1543–1554. 10.3382/ps/pew088 26976906

[B47] Su Y. Yao W. Perez-Gutierrez O. N. Smidt H. Zhu W. Y. (2008). Changes in abundance of *Lactobacillus spp*. and *Streptococcus suis* in the stomach, jejunum and ileum of piglets after weaning. *FEMS Microbiol. Ecol.* 66 546–555. 10.1111/j.1574-6941.2008.00529.x 18554303

[B48] Suiryanrayna M. V. Ramana J. V. (2015). A review of the effects of dietary organic acids fed to swine. *J. Anim. Sci. Biotechnol.* 6:45. 10.1186/s40104-015-0042-z 26500769 PMC4618844

[B49] Takahashi N. Yamada T. (1999). Glucose and lactate metabolism by *Actinomyces naeslundii*. *Crit. Rev. Oral Biol. Med.* 10 487–503. 10.1177/10454411990100040501 10634585

[B50] Tsiloyiannis V. K. Kyriakis S. C. Vlemmas J. Sarris K. (2001). The effect of organic acids on the control of porcine post-weaning diarrhoea. *Res. Vet. Sci.* 70 287–293. 10.1053/rvsc.2001.0476 11676629

[B51] Yang Y. X. Mu C. L. Zhang J. F. Zhu W. Y. (2014). Determination of biogenic amines in digesta by high performance liquid chromatography with precolumn dansylation. *Anal. Lett.* 47 1290–1298. 10.1080/00032719.2013.871550

[B52] Yano J. M. Yu K. Donaldson G. P. Shastri G. G. Ann P. Ma L. (2015). Indigenous bacteria from the gut microbiota regulate host serotonin biosynthesis. *Cell* 161 264–276. 10.1016/j.cell.2015.02.047 25860609 PMC4393509

[B53] Yu K. F. Mu C. L. Yang Y. X. Su Y. Zhu W. Y. (2017). Segment-specific responses of intestinal epithelium transcriptome to in-feed antibiotics in pigs. *Physiol. Genomics* 49 582–591. 10.1125/physiolgenomics.00020.2017 28887368

[B54] Yu M. Mu C. L. Yang Y. X. Zhang C. J. Su Y. Huang Z. (2017a). Increases in circulating amino acids with in-feed antibiotics correlated with gene expression of intestinal amino acid transporters in piglets. *Amino Acids* 49 1587–1599. 10.1007/s00726-017-2451-0 28623466

[B55] Yu M. Zhang C. J. Yang Y. X. Mu C. L. Su Y. Yu K. F. (2017b). Long-term effects of early antibiotic intervention on blood parameters, apparent nutrient digestibility, and fecal microbial fermentation profile in pigs with different dietary protein levels. *J. Anim. Sci. Biotechnol.* 8:60. 10.1186/s40104-017-0192-2 28781770 PMC5537924

[B56] Zhang L. L. Mu C. L. He X. Y. Su Y. Mao S. Y. Zhang J. (2016). Effects of dietary fibre source on microbiota composition in the large intestine of suckling piglets. *FEMS Microbiol. Lett.* 363:fnw138. 10.1093/femsle/fnw138 27231242

[B57] Zoetendal E. G. Akkermans A. D. L. Vos W. M. D. (1998). Temperature gradient gel electrophoresis analysis of 16S rRNA from human fecal samples reveals stable and host-specific communities of active bacteria. *Appl. Environ. Microbiol.* 64 3854–3859. 9758810 10.1128/aem.64.10.3854-3859.1998 PMC106569

