# Supplementary material for: Marked Response in Microbial Community and Metabolism in the Ileum and Cecum of Suckling Piglets After Early Antibiotics Exposure
Source: Front Microbiol. 2018 May 30;9:1166. doi: 10.3389/fmicb.2018.01166 (PMC5989621; doi:10.3389/fmicb.2018.01166)

**Marked Response in Microbial Community and Metabolism in the Ileum and Cecum of Suckling Piglets after Early Antibiotics Exposure**

Miao Yu^a,b,c^, Chunlong Mu^a,b^, Chuanjian Zhang^a,b^, Yuxiang Yang^a,b^, Yong Su^a,b^ and Weiyun Zhu^a,b†^

^a^ Jiangsu Key Laboratory of Gastrointestinal Nutrition and Animal Health, Laboratory of Gastrointestinal Microbiology, College of Animal Science and Technology, Nanjing Agricultural University, Nanjing 210095, China

**^b^** National Center for International Research on Animal Gut Nutrition, Nanjing Agricultural University, Nanjing 210095, China

**^c^** Guangdong Key Laboratory of Animal Breeding and Nutrition, State Key Laboratory of Livestock and Poultry Breeding, Institute of Animal Science, Guangdong Academy of Agricultural Sciences, Guangzhou 510640, China

**Supplementary material**

**Summary**

The supporting information includes 2 supplementary table and 3 supplementary figures.

Table S1. Ingredient and nutrient composition of creep feed (%, as-fed basis)^1^

| Ingredients (%) | |
| --- | --- |
| Corn | 40.00 |
| Rice, broken | 15.00 |
| Soybean meal, fermented | 10.00 |
| Soybean meal, de-hulled | 6.00 |
| Spray dried animal plasma | 5.00 |
| Whey powder | 7.00 |
| Fish meal | 4.00 |
| Sugar | 4.50 |
| Glucose | 3.00 |
| Soybean oil | 1.50 |
| L-Lysine-HCl (98%) | 0.30 |
| L-Methionine | 0.15 |
| L-Threonine | 0.20 |
| L-Tryptophan | 0.05 |
| L-Isoleucine | 0.05 |
| L-Valine | 0.05 |
| Sodium chloride | 0.30 |
| Limestone | 1.10 |
| CaHPO4 | 0.80 |
| Vitamin mixture^1^ | 0.20 |
| Mineral mixture^2^ | 0.80 |
| Total | 100.00 |
| Nutrient composition, % |  |
| Crude protein | 20.20 |
| Digestible energy (Mcal/kg) | 3.40 |
| Total calcium | 0.85 |
| Total phosphorus | 0.70 |
| Digestible Lys | 1.45 |
| Digestible Met+Cys | 0.79 |
| Digestible Thr | 0.81 |
| Digestible Trp | 0.23 |
| Digestible Ile | 0.74 |
| Digestible Leu | 1.45 |
| Digestible Val | 0.89 |
| Analyzed nutrient composition, % |  |
| Crude protein | 20.13 |

^1^ Vitamin mixture supplied the following per kg complete diet: vitamin A, 15,000 IU; vitamin D3, 3,000 IU; vitamin E, 150 mg; vitamin K3, 3 mg; vitamin B1, 3 mg; vitamin B2, 6 mg; vitamin B6, 5 mg; vitamin B12, 0.03 mg; niacin, 45 mg; vitamin C, 250 mg; calcium pantothenate, 9 mg; folic acid, 1 mg; biotin, 0.3 mg; choline chloride, 500 mg.

Mineral mixture supplied the following per kg complete diet: Fe, 170 mg; Cu, 150 mg; I, 0.90 mg; Se,0.2 mg; Zn, 150 mg; Mg, 68 mg; Mn, 80 mg; Co, 0.3 mg.

Table S2. Ingredient and nutrient composition of Sow’ diet (%, as-fed basis)^1^

| Ingredients (%) | |
| --- | --- |
| Corn | 70.00 |
| Soybean meal | 20.00 |
| Fish meal | 2.00 |
| Wheat bran | 4.00 |
| Premix ^1^ | 4.00 |
| Total | 100.00 |
| Nutrient composition, % |  |
| Crude protein | 17.50 |
| Digestible energy (Mcal/kg) | 3.12 |
| Crude fatty | 3.07 |
| Crude Fiber  Total calcium | 2.05  0.77 |
| Digestible Lys | 0.88 |
| Digestible Met+Cys | 0.67 |

^1^ Premix for the sows provided following per kg complete diet: vitamin A, 10,000 IU; vitamin D3, 1,100 IU; vitamin E, 30 mg; vitamin K3, 1.4 mg; vitamin B1, 1.5 mg; vitamin B2, 3 mg; vitamin B6, 2.5 mg; vitamin B12, 0.02 mg; niacin, 20 mg; choline chloride, 0.31 mg, Fe, 150 mg; Cu, 140 mg; I, 0.12 mg; Se,0.27 mg; Zn, 100 mg.

Figure S1 Rarefaction curves comparing the number of sequences with the number of phylotypes found in the 16S rRNA gene libraries from the microbiota in the digesta of the ileum and cecum of piglets in the control and antibiotics group.

Figure S2 Influence of early antibiotics exposure on the 30 most abundant genera in the ileum. The color represents the relative abundance of bacteria.


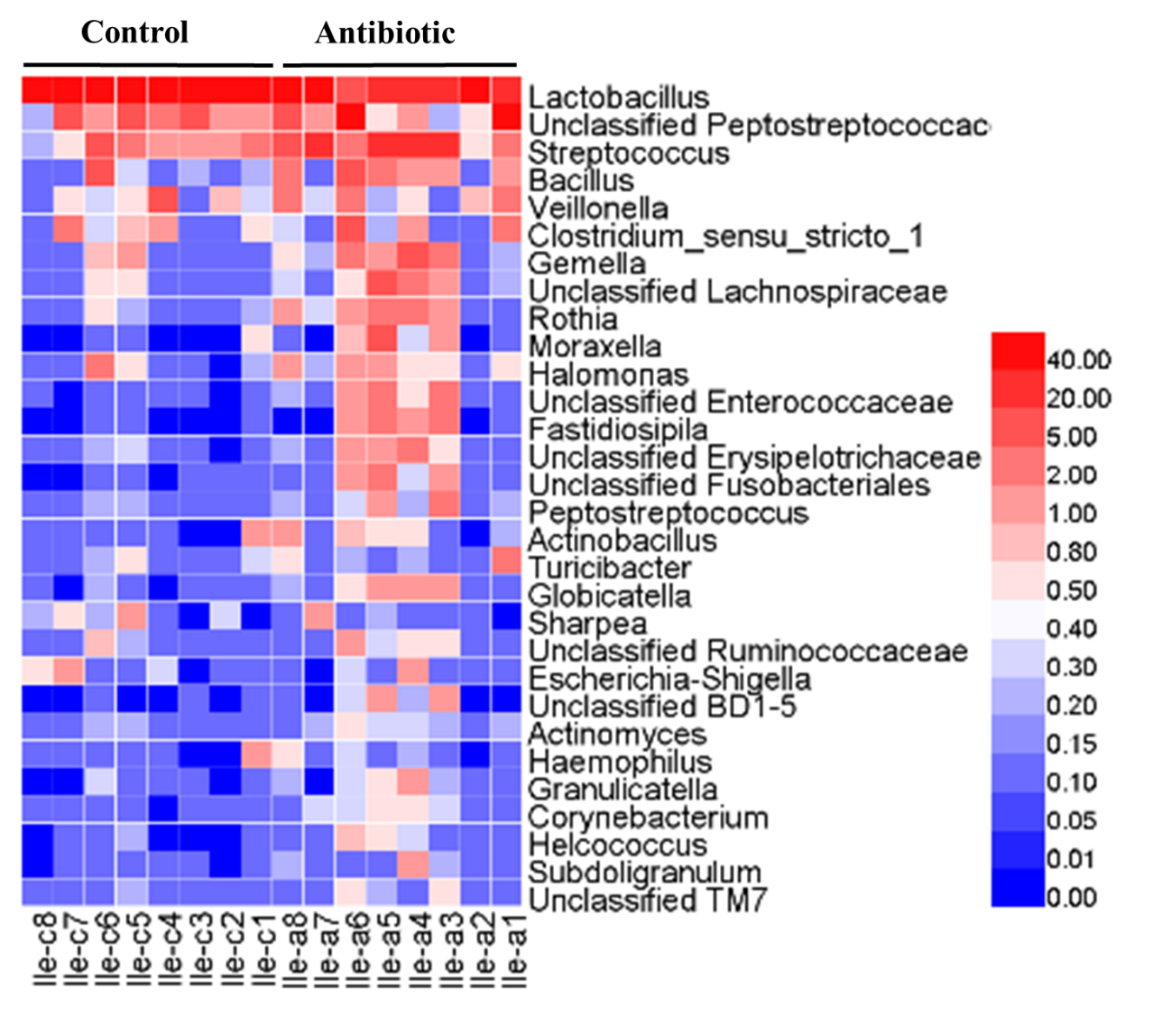


Figure S3 Influence of early antibiotic exposure on the 30 most abundant genera in the cecum. The color represents the relative abundance of bacteria.


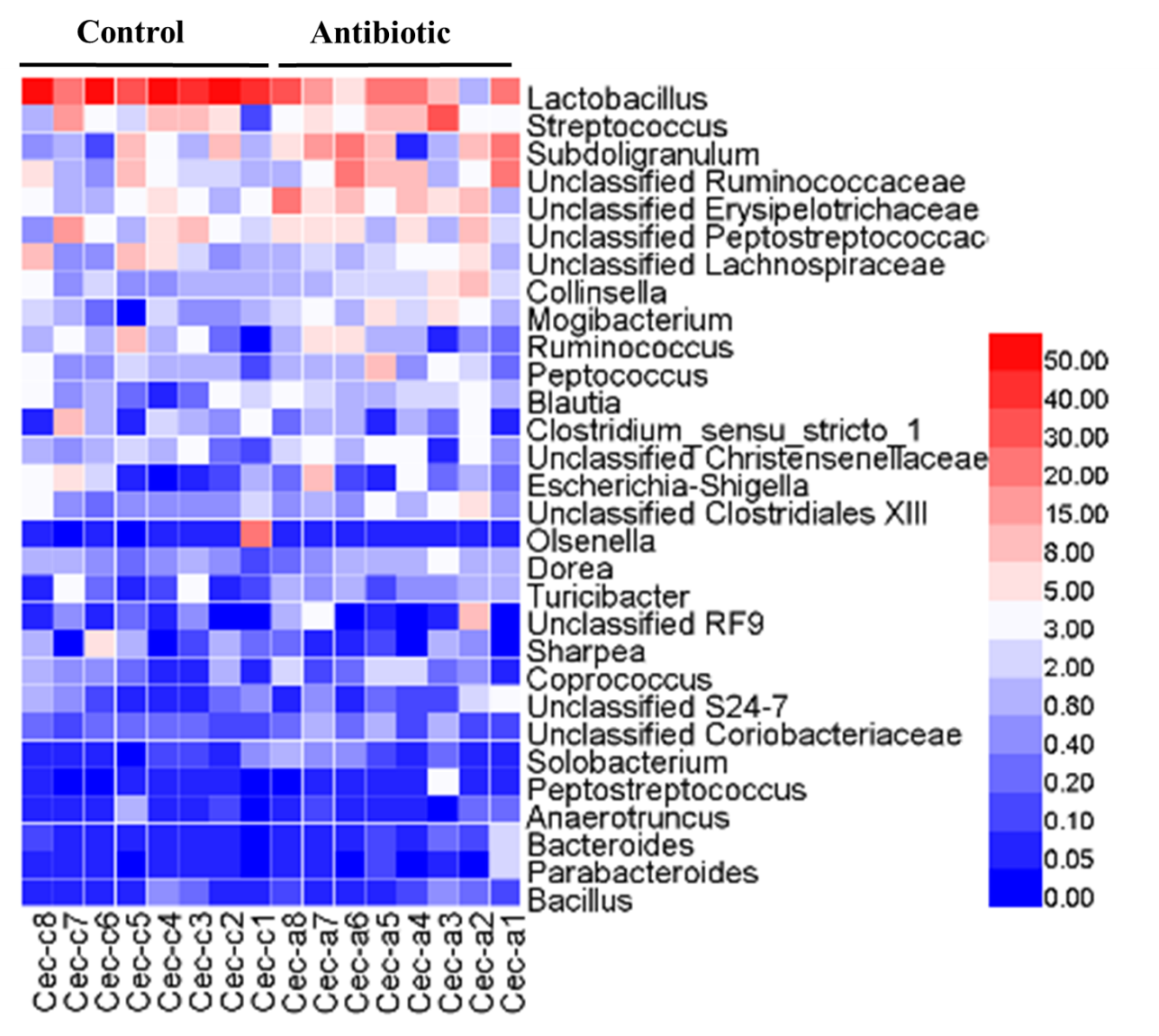

Supplement: Supplementary file 1 [file Data_Sheet_1.docx]
